# Effects of a Reserve Protein on *Spodoptera frugiperda* Development: A Biochemical and Molecular Approach to the Entomotoxic Mechanism

**DOI:** 10.3390/molecules25092195

**Published:** 2020-05-08

**Authors:** Carolina Turatti Oliveira, Suzy Wider Machado, Cézar da Silva Bezerra, Marlon Henrique Cardoso, Octávio Luiz Franco, Carlos Peres Silva, Demetrio Gomes Alves, Cristina Rios, Maria Lígia R. Macedo

**Affiliations:** 1 Faculdade de Ciências Farmacêuticas, Alimentos e Nutrição, Universidade Federal de Mato Grosso do Sul, Campo Grande, Mato Grosso do Sul 79070-900, Brazil; caroltuo@hotmail.com (C.T.O.); suzywider@gmail.com (S.W.M.); 2 Programa multicêntrico de Pós-Graduação em Bioquímica e Biologia Molecular, Instituto de Biologia, Universidade Federal de Mato Grosso do Sul, Campo Grande, Mato Grosso do Sul 79070-900, Brazil; cezar.bezerra@gmail.com; 3 Centro de Análises Proteômicas e Bioquímicas, Pós-Graduação em Ciências Genômicase Biotecnologia, Universidade Católica de Brasília, Brasília, Distrito Federal 70790-160, Brazil; marlonhenrique6@gmail.com (M.H.C.); ocfranco@gmail.com (O.L.F.); 4 S-Inova Biotech, Programa de Pós-Graduação em Biotecnologia, Universidade Católica Dom Bosco, Campo Grande, Mato Grosso do Sul 79117-900, Brazil; 5 Departamento de Bioquímica, Centro de Ciências Biológicas, Universidade Federal de Santa Catariana, Florianópolis, Santa Catarina 88040-900, Brazil; carlos.peres@ufsc.br (C.P.S.); demetrio.gomes@gmail.com (D.G.A.); crisaqi1@gmail.com (C.R.)

**Keywords:** multifunctional protein, lectin properties, bioinsecticides, enzyme activity, insect gut

## Abstract

Talisin is a storage protein from *Talisia esculenta* seeds that presents lectin-like and peptidase inhibitor properties. These characteristics suggest that talisin plays a role in the plant defense process, making it a multifunctional protein. This work aimed to investigate the effects of chronic intake of talisin on fifth instar larvae of *Spodoptera frugiperda*, considered the main insect pest of maize and the cause of substantial economic losses in several other crops. The chronic intake of talisin presented antinutritional effects on the larvae, reducing their weight and prolonging the total development time of the insects. In addition, talisin-fed larvae also showed a significant reduction in the activity of trypsin-like enzymes. Midgut histology analysis of talisin-fed larvae showed alterations in the intestinal epithelium and rupture of the peritrophic membrane, possibly causing an increase of aminopeptidase activity in the midgut lumen. Talisin also proved to be resistant to degradation by the digestive enzymes of *S. frugiperda*. The transcription profile of trypsin, chymotrypsin and aminopeptidase genes was also analyzed through qPCR technique. Talisin intake resulted in differential expression of at least two genes from each of these classes of enzymes. Molecular docking studies indicated a higher affinity of talisin for the less expressed enzymes.

## 1. Introduction

Insect pest control is one of the major problems facing agriculture because of the need for agrochemicals to be more environmentally sound, economically efficient and not pose a threat to human health. Different strategies have to be adopted to optimize crop productivity, mainly considering the rapid world population growth [1]. Cultivars expressing plant defense genes have shown efficient results against insects, pointing toward a promising direction. Insects fed on transformed plants exhibit remarkable effects on larval weight, fecundity, survival and, consequently, lower plant damage [2,3,4]. However, the development of insect resistance against single-defense genes, including those currently used in *Bt-crops,* is well known [5,6].

Talisin is a *Talisia esculenta* seed protein that presents both peptidase inhibitors and lectin-like properties [7], which consist of two classes of proteins involved in plant defense mechanisms [8]. Previous studies showed that talisin intake promotes insecticidal activity against diverse insects [9,10,11]. In parallel, insects are one of the most evolutionarily well-adapted groups of organisms worldwide, mainly due to their ability to exploit diverse habitats and food sources, which may have induced the evolution of a large number of digestion enzymes [12].

The fall armyworm, *Spodoptera frugiperda* (Lepidoptera: Noctuidae), is one of the insect pests responsible for considerable damage to various crops around the world, due to its generalist habit [13]. One of the main insect control strategies is the use of transgenic crops, most of which were modified to carry the *Bt* (*Bacillus thuringiensis*) gene that codes for a particular endotoxin. There is an obvious need to better understand how plant bioactive compounds interact and affect insects, as this knowledge would help to uncover the causes of insect pests’ development and their resistance mechanisms. A very promising alternative seems to be the expression of proteins with different modes of action [14]. In this context, this work aimed to explore the multifunctional properties of talisin, focusing on the investigation of talisin intake effects on *S. fugiperda* development and on their main digestive enzymes’ activity, as well as on their expression pattern. Moreover, structural and histopathology studies were also explored.

## 2. Results

### 2.1. Effects of talisin on Insect Development

**Figure 1:**
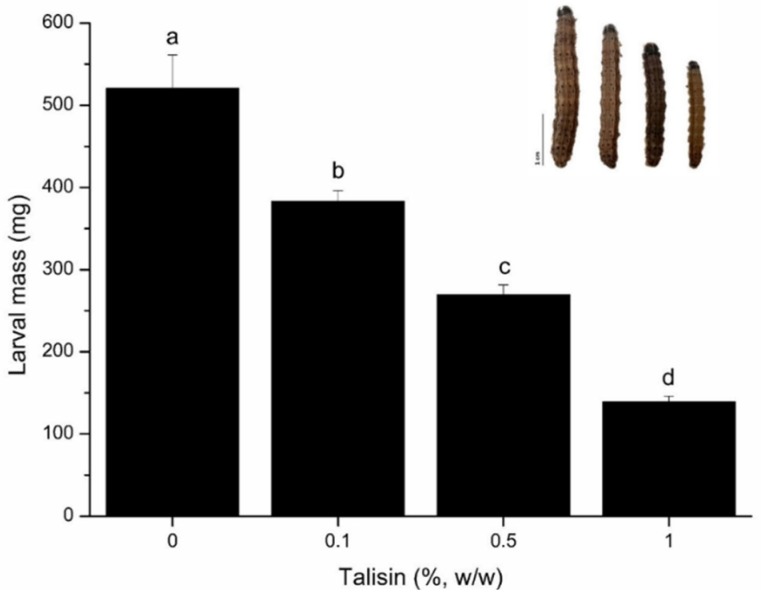
Effect of dietary *Talisia esculenta* reserve protein (talisin) on mass of fifth-instar *Spodoptera frugiperda* larvae. Inset: size difference in a control larva (left) and a larva fed 0.1, 0.5 and 1% talisin (right). Different letters indicate significant differences (*p* < 0.05; Tukey’s test). Bar = 1 cm.

Diets containing 0.1%, 0.5%, and 1% talisin (*w*/*w*) caused fifth-instar larval mass to decrease by about 26, 48 and 73%, respectively (Figure 1). Despite this significant weight reduction (*p* < 0.05), talisin intake did not affect larval survival.

In talisin-fed larvae, the larval stage was extended by 1 day and the total development time, which comprises hatching to adult death, was 3.3 days longer than the control (Table 1). Furthermore, no significant differences were found in the physiological parameters analyzed in the larvae fed with talisin 0.5% (Table 1).

### 2.2. Nutritional Data

Talisin-fed larvae consumed 9% more food and produced 36% less frass than controls, resulting in a significant difference in the approximate digestibility (AD) (Table 2). AD is an index that indicates the percentage of ingested food that is effectively assimilated by the insect and in talisin-fed larvae AD increased by 21%. No other nutritional parameter was changed (Table 2).

### 2.3. Digestive Enzyme Activity in Larvae Fed on Talisin-Amended Diet

**Figure 2:**
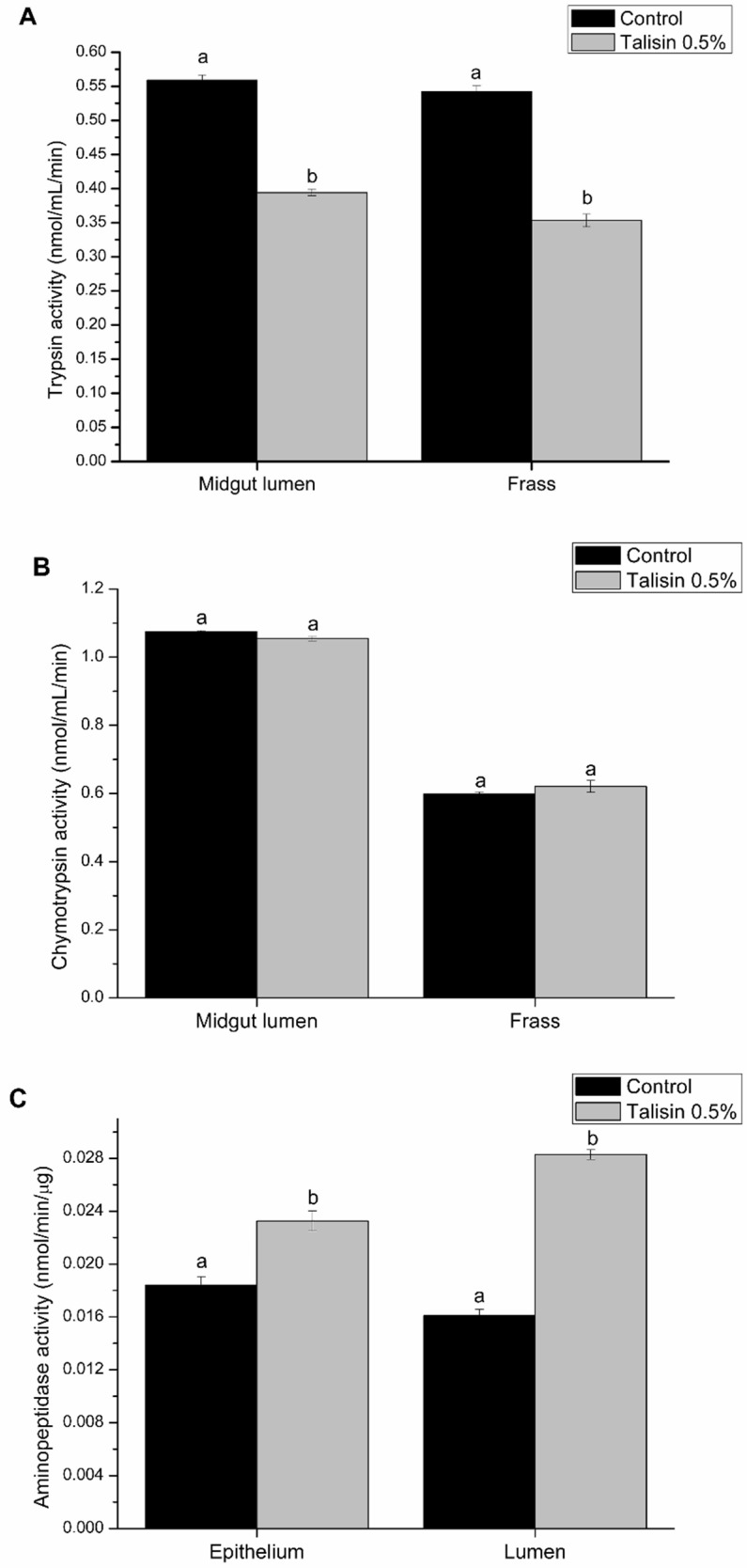
Trypsin-like (**A**), chymotrypsin-like (**B**) and aminopeptidase (**C**) activities in control and experimental (0.5% talisin-fed) *Spodoptera frugiperda* larvae. Different letters indicate significant differences (*p* < 0.05; Tukey’s test).

**Figure 3:**
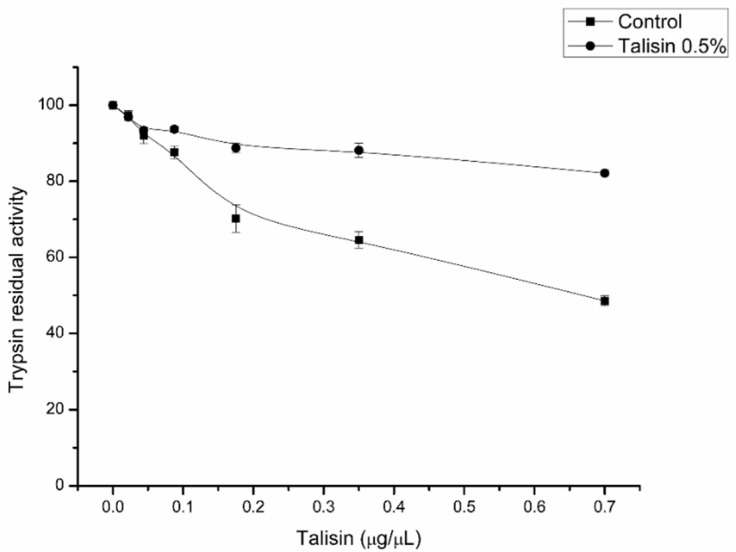
In vitro inhibition of proteolytic activity of midgut trypsins of *Spodoptera frugiperda* larvae fed on control diet (square) and diet containing 0.5% talisin (circle), using the synthetic BApNA substrate.

Differences in food consumption and utilization were followed by differences in digestive enzyme activity. The in vitro proteolytic activity of trypsin-like enzymes, both in the midgut lumen and in the frass, decreased by 30% and 35%, respectively (Figure 2A). Furthermore, sensitivity of trypsin-like enzymes present in the midguts from talisin-fed larvae was markedly decreased (Figure 3). Aminopeptidase-*N* activity from the epithelial sample of talisin-fed larvae increased by 25% compared to the controls. This increase was even more significant in samples from the midgut lumen, about 70% (Figure 2C). No differences were observed in the chymotrypsin-like (Figure 2B) and α-amylase activities.

### 2.4. In-Gel Visualization of Peptidase Activity

**Figure 4:**
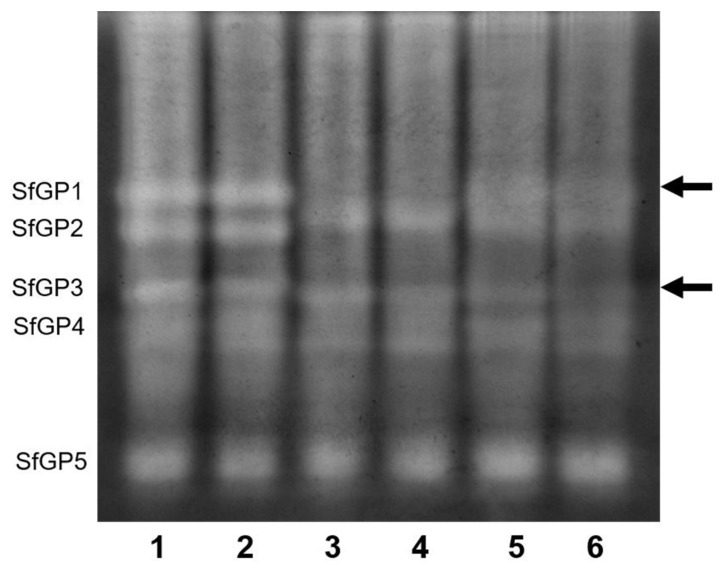
Peptidase activity of midgut peptidases in zymography gel electrophoresis. Samples fed with control diet or diet containing 0.5% talisin were used for the determination of enzyme profile. The bands were designated SfGP1 through SfGP5. Lane 1 was loaded with midgut juice extract from larvae fed on a control diet devoid of talisin; lane 2, with midgut juice extract from larvae fed on a 0.5% talisin diet; lanes 3 and 5, with midgut juice extract from larvae-fed control diets mixed with TLCK and talisin, respectively; lanes 4 and 6 with midgut juice extract from larvae fed 0.5% talisin diets mixed with TLCK and talisin, respectively.

Casein zymography was employed for visualization of proteinase activity using midgut lumen of control and 0.5% talisin-fed larvae. *S. frugiperda* gut peptidases (SfGP) were designated SfGP1 through SfGP5 (Figure 4). Incubation of samples with TLCK suggested that SfGP1 and SfGP3 are trypsin-like enzymes. The comparison of activities between control and experimental larvae (lines 1 and 2) revealed no differences among the bands, suggesting that major SfGP are similar in both control and talisin-fed larvae (Figure 4, lanes 1 and 2). Talisin incubation with midgut homogenates, both from control and talisin-fed larvae, showed that the peptidase activity was subtly inhibited (Figure 4, right-side arrow, lanes 5 and 6). 

### 2.5. Talisin is Resistant to Hydrolysis by Midgut Peptidase

**Figure 5:**
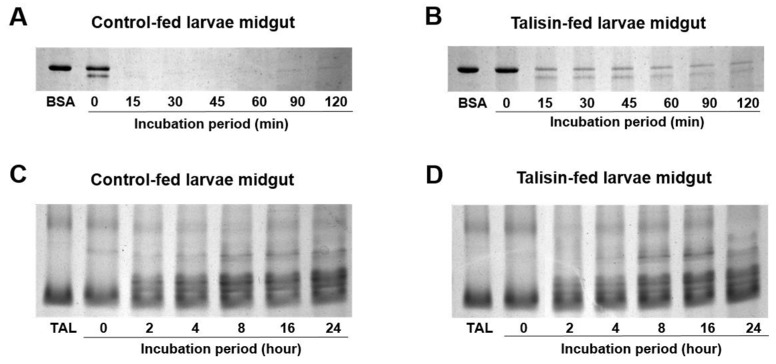
Resistance of talisin (*Talisia esculenta* reserve protein) to hydrolysis by *Spodoptera frugiperda* midgut peptidases. SDS–PAGE of midgut juice extract mixed with talisin at a 1:5 (lectin to midgut homogenates) ratio and incubated at 30 °C for different periods. Digestion of BSA (**A**) and talisin (**C**) with midgut juice extract of control-fed larvae and digestion of BSA (**B**) and talisin (**D**) with midgut juice extract of talisin-fed larvae. TAL: talisin; BSA: bovine serum albumin; MG: midgut.

To evaluate whether talisin is resistant to degradation by peptidases present in the larval midgut, talisin was mixed with midgut juice extract of control-fed and talisin-fed larvae and incubated for periods of up to 24 h. SDS-PAGE demonstrated that the band with apparent molecular weights of 22.1 kDa, corresponding to talisin, remained in the samples after 24 h of incubation, indicating a strong talisin resistance to proteolysis by midgut peptidases (Figure 5C,D). SDS-PAGE performed with BSA showed that the enzymes from control-fed larvae were active and capable of degrading BSA within 15 min, whereas enzymes from talisin-fed larvae midgut had a longer delay to be able to degrade the BSA (Figure 5A,B).

### 2.6. Microscopy Analysis

**Figure 6:**
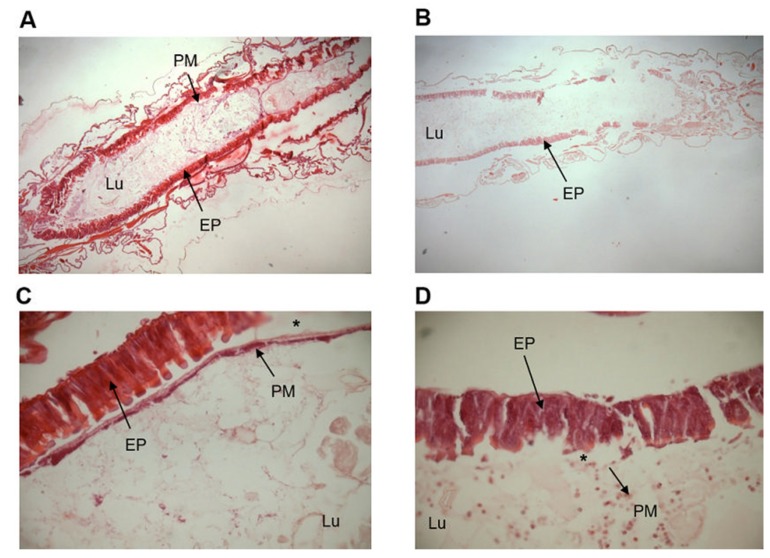
Photomicrographs of fifth-instar larvae of *Spodoptera frugiperda*. (**A**) Midgut of control larvae, 10×. (**B**) Midgut of larvae fed on a 0.5% talisin diet, 10× (**C**) Midgut epithelium of control larvae, 40× (**D**) Midgut epithelium of larvae fed on a 0.5% talisin diet, 40×. Lu, Lumen; EP, Epithelium; PM, Peritrophic Membrane; (*) Difference between the ectoperitrophic space.

Due to the differences between the aminopeptidase-*N* activity in the lumen and in the epithelium samples, we investigated the integrity of the midgut membranes. The midgut epithelial layer of control larvae presented a single layer of columnar cells and the integrative peritrophic membrane (Figure 6A,C). In talisin-fed larvae, it is possible to notice changes in the epithelial cells, the less thick peritrophic membrane with ruptures, and a reduction in ectoperitrophic space (Figure 6B,D).

### 2.7. Real-Time PCR

**Figure 7:**
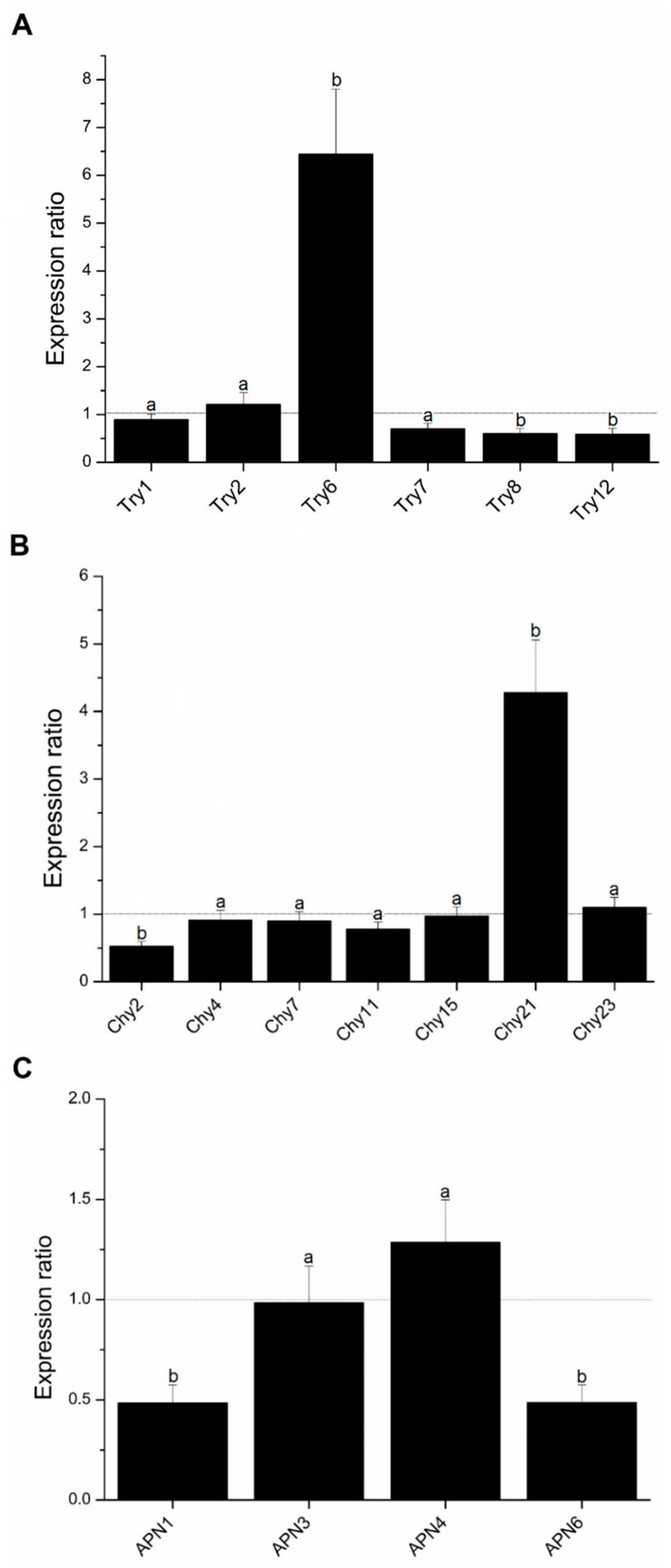
Relative expression of trypsin (**A**), chymotrypsin (**B**) and *N*-aminopeptidase (**C**) genes of fifth instar of talisin-fed *Spodoptera frugiperda* larvae. Genes above the dotted line were considered more expressed and genes below the dotted line were considered less expressed. Different letters indicate significant differences (*p* < 0.05).

Three out of the six trypsin genes analyzed showed a differential expression in talisin-fed larvae. The Try6 gene showed an increase in relative expression up to 6-fold. The Try8 and Try12 genes were less expressed (Figure 7A). Regarding the chymotrypsin genes, only two genes presented differential expression. Chy21 saw its expression increased by 4-fold, and the Chy2 gene was less expressed (Figure 7B). The other genes, both trypsin and chymotrypsin, did not present differential transcription when compared to talisin intake. However, the APN1 and APN6 genes, related to *N*-aminopeptidases, were less expressed after talisin ingestion, and the others did not present differential transcription with respect to talisin intake (Figure 7C).

### 2.8. Molecular Modeling and Docking

In order to verify the interactions and affinity between talisin and the more/less expressed enzymes observed in the real-time PCR, these proteins were modeled and further submitted to docking studies to shed light on the talisin-enzyme complexes. Talisin presented the best coverage (92%) and identity (42%) values allied to the low E-value when aligned to the primary sequence of a *Delonix regia* Kunitz-type serine peptidase inhibitor (PDB id: 1R8N). For trypsin 6 and 12, the crystallographic structure of *Fusarium oxysporum* (PDB id: 1gdu) was selected as the model structure of atomic coordinates. For chymotrypsin 2 and 21, the model structure used was a collagenase from the larvae of the *Hypoderma lineatum* (PDB id: 1hyl) fly. The ProSa-web analyses also confirmed the fold quality of the theoretical models obtained, revealing equivalent z-scores (−4.62, −5.82, −5.28, −5.42 and −5.82 for talisin, Try6, Try12, Chy2 and Chy21, respectively) of proteins structurally resolved by X-ray crystallography and deposited in the Protein Data Bank (PDB). All validated models presented above 87% of the residues in the most favorable regions of the Ramachandran plot. For detailed information on all structure statistics, please see Table 3. All these characteristics and parameters revealed the reliability of the constructed models, making them suitable for molecular docking studies.

**Figure 8:**
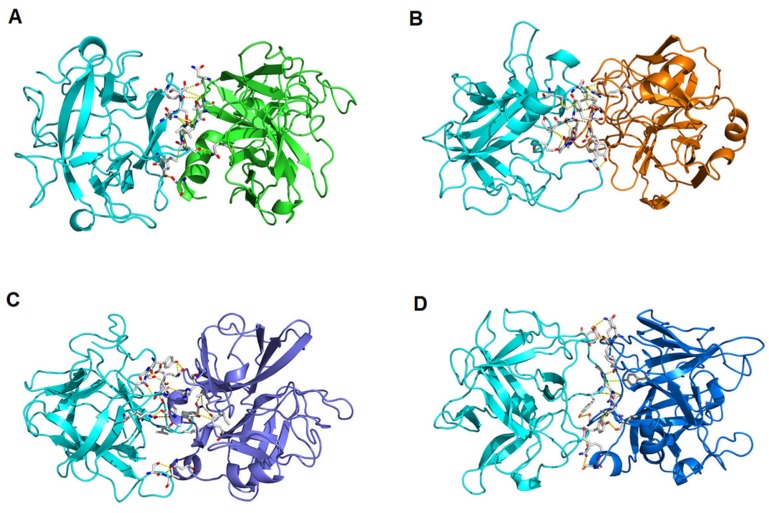
Theoretical three-dimensional model for the talisin/Try 6 (**A**), talisin/Try 12 (**B**), talisin/Chy 6 (**C**) and talisin/Chy 12 (**D**) complexes.

The best affinity values for the talisin/Try 6, talisin/Try 12, talisin/Chy 2 and talisin/Chy 21 complexes were −8.4 −8.8, −11.2, −10.0 kcal·mol^−1^, respectively. Therefore, we observed that talisin has a slightly greater affinity for Trypsin 12 (less expressed), than for Trypsin 6 (more expressed). Talisin also shows higher affinity for Chymotrypsin 2 (less expressed) than for Chymotrypsin 21 (most expressed). These data corroborate our in vitro findings regarding the relative expression of the genes coding for these enzymes. The atomic interactions of the talisin/Try 6 complex were predicted to range from 1.9 to 3.6 Å, consisting of 9 hydrogen bonds and 1 hydrophobic interaction. The talisin/Try 12 complex had 12 hydrogen bonds and 2 hydrophobic interactions, where the distances between all atoms involved in interactions ranged from 1.7 to 3.6 Å (Table 4, Figure 8A,B). The talisin/Chy 2 complex presented 13 hydrogen bonds and 3 hydrophobic interactions, varying from 2.6 to 3.6 Å distance. Finally, the talisin/Chy 21 complex had the same number of hydrogen bonds as the talisin/Chy 2 complex and only one hydrophobic interaction, varying from 2.9 to 3.6 Å (Table 5, Figure 8C,D).

## 3. Discussion

Talisin presents high similarity with several storage proteins [7], and also presents both peptidase inhibitor and lectin-like properties, which comprise two classes of proteins involved in plant defense mechanisms and that can lead to toxic effects when ingested by insects [8,18,19]. Therefore, talisin can be considered a multifunctional protein, and such versatility makes this protein worth exploring for its insecticidal potential.

In order to evaluate the in vivo effect of talisin ingestion by fifth instar larvae of *S. frugiperda*, an artificial diet was offered to the larvae with different amounts of talisin. The weight reduction indicates that talisin causes an antinutritional effect, hindering the absorption of nutrients. Although talisin causes a significant decrease in larval mass, we did not observe a change in survival rate of the larvae, possibly due to talisin impairing but not completely blocking the digestion of proteins, allowing the insect to develop mechanisms that enable it to survive and adapt [20,21]. Macedo, Freire, Kubo and Parra [10] reported a reduction of 50 and 76% in the weight of *Anticarsia gemmatalis* larvae fed on an artificial diet containing 1.5 and 2.0% (*w*/*w*) of talisin, respectively, and also observed no change in survival rate or the time of larval development.

Incorporation of 0.5% talisin into an artificial diet decreased larval mass by approximately 50%. This concentration corresponds to lectin and other plant defense protein levels present in legume seeds and is similar to that employed in other studies [22,23]. In addition to affecting the larval mass gain, along with higher food intake and lower feces production, talisin intake caused alterations in the approximate digestibility (AD) (Table 2). AD is an index that indicates the percentage of ingested food that is effectively assimilated by the insect, or more specifically by the walls of the insect’s gut. The higher food retention in the midgut is an attempt to maximize AD, probably to meet the increased demand for nutrients and to compensate for the antinutritional effect of talisin [24].

Talisin ingestion also led to changes in the insect development period, with a 1-day increase in larval stage and 3.3 days more in total development time (TDT) (Table 1). Similarly, Li and Romeis [25] reported a 3.7-day increase in TDT from *Chrysoperla carnea* (Neuroptera) fed 1% GNA, a well-studied lectin, purified from *Galanthus nivalis* (Amaryllidaceae). This delay in larval development has also been observed *in Helicoverpa armigera* and *Spodoptera litura* when fed with miraculin-like proteins, with which talisin presents amino acid sequence similarity (50%) [26]. The authors reported a 7-day increase in the TDT of *H. armigera* larvae after ingestion of 0.43% *Murraya koenigii* miraculin-like protein (MKMLP). Slowing down larval growth, especially in earlier larval stages, makes lepidopterans vulnerable to predators for a longer period—which in economic terms could be an advantage.

For plant proteins to effectively exert an antinutritional effect when ingested by insects they must be resistant to the action of the peptidases from the insect’s digestive tract [27,28]. Otherwise the protein can be cleaved, losing its insecticidal activity [29]. To evaluate talisin’s resistance to the action of *S. frugiperda* peptidases, it was incubated for up to 24 h with the intestinal extract of the larvae. Through a PAGE-SDS, we verified that the midgut peptidases from *S. frugiperda* are incapable of degrading talisin, which is resistant to proteolysis for up to 24 h, the period during which digestive enzymes remain active (Figure 5C). Interestingly, BSA, which was used as a positive control, is completely degraded by the peptidases from larvae fed on a control diet in a short period of time (15 min) (Figure 5A), whereas the peptidases from larvae fed on a talisin diet degraded BSA only partially and in a much slower way (Figure 5B). These findings support the fact that talisin interferes in the proteolytic activity of this insect inhibiting the activity of digestive enzymes. The digestibility of plant reserve proteins is related to the exceptionally high structural stability (Xia et al., 2016). Our results corroborate previous studies showing the high resistance of talisin to proteolysis by digestive enzymes of insects, since this protein has already been resistant to degradation by the peptidases of *Anticarsia gemmatalis* [10], *Callosobruchus maculatus* [30], and *Diatraea saccharalis* [9].

Some insects from the order Lepidoptera and Coleoptera have great ability to alter digestive peptidases in response to qualitative nutritional changes in the diet and/or when existing peptidases are ineffective and/or inefficient for digestion [20,31,32,33,34,35]. One of these mechanisms includes increased activity of intestinal peptidases to achieve the optimal rate of protein digestion [36,37]. The PAGE-SDS gel with the midgut extracts of talisin-fed larvae indicates that the proteolytic enzymes of these insects were unable to degrade talisin (Figure 5). 

In a previous study, Freire, Vasconcelos, Oliveira, Filho and Macedo [7] performed the biochemical characterization and cloning of talisin, which showed a high similarity with several reserve proteins, all of which presented amino acid sequences clearly related to the Kunitz family of peptidase inhibitors (family I3 on MEROPS database). Considering this peptidase inhibitor property, along with the fact that lepidoptera such as *S. frugiperda* have the serinepeptidases trypsin and chymotrypsin as the main enzymes responsible for protein digestion [38], the first attempt involved investigating the in vivo effect of ingestion of talisin on these two enzymes. Trypsin-like enzymes from the midgut lumen of larvae chronically fed on talisin presented a reduction of 30% when compared to the control larvae. A similar reduction (about 33%) was also observed in feces. 

We employed the zymography using casein as substrate to visualize the major bands with proteolytic activity in *S. frugiperda* midgut (Figure 4). Since trypsin is the major peptidase involved in larval digestion, we further incubated the midgut extracts with the irreversible trypsin inhibitor TLCK. Finally, we incubated the midgut extracts with talisin to understand the effects of Talisin on bands with proteolytic activity. The zymography showed the presence of five bands, named *S. frugiperda* gut peptidases (SfGP), from SfGP1 to SfGP5. The comparison of activities between control and experimental larvae (lines 1 and 2) revealed no differences among the bands, suggesting that major SfGP are similar in both control and talisin-fed larvae. The incubation of TLCK in control and talisin-fed larvae samples prompted a strong inhibition of SfGP1 and SfGP3, suggesting that these band correspond to trypsin activity. The incubation of talisin with midgut homogenates (lines 5 and 6), revealed the in both control and talisin-fed larvae the SfGP1 was partially inhibited, in an intensity intermediate between the samples with (lines 3 and 4) and without (lines 1 and 2) TLCK. We also noticed that in talisin-fed larvae the SfGP3 was partially inhibited. Thus, we showed that the incubation of talisin inhibited the proteolytic activity of SfGP1 and SfGP3, bands inhibited by TLCK, suggesting that talisin showed inhibition against trypsin enzymes in zymography (right-side arrow in Figure 4).

Chymotrypsin activity was not altered either in the lumen or in the feces (Figure 2). Stevens, Dunse, Guarino, Barbeta, Evans, West and Anderson [20] detected a high loss of peptidases in *Helicoverpa armigera* feces fed with the NaPI serine peptidase inhibitor and suggested that this large amount of lost enzymes limits the amount of enzymes that can be recycled by the insect, reducing the pool of amino acids and nitrogen for the synthesis of proteins that, consequently, leads to a reduction in larval growth. It is possible that talisin, when bound to the digestive enzymes of the midgut, forming a complex, may prevent the reabsorption of these enzymes from the endoperitrophic space into the ectoperitrophic space, causing a critical loss of essential amino acids for the feces. Furthermore, because it is not degraded as it goes through the digestive tract of *S. frugiperda*, talisin is possibly being completely eliminated in the feces (data not shown), which corroborates the reduction of trypsin activity in feces.

In addition, we found that trypsins from talisin-fed larvae had a lower inhibition by talisin (Figure 3). This result suggests that talisin-fed larvae break down proteins with trypsins that are less sensitive to inhibition by talisin. This apparent difference in inhibition may arise from the differential expression of enzymes, since one of the most common mechanisms in lepidopteran species (e.g. *S. frugiperda*) in response to the ingestion of toxic proteins (e.g. peptidase inhibitors) is the overproduction of peptidases or the expression of a new peptidase that is insensitive to peptidase inhibitors [31,36]. Also, it would not be possible to verify the production of isoforms via zymography, since most isoforms present modifications of a few amino acids, and this could not be visualized in gel. Thus, we sought to verify differences in the gene expression of enzymes through qPCR.

Through the expression analyses of trypsin and chymotrypsin genes, we observed that talisin intake causes a differential expression of only three trypsin genes and two chymotrypsin genes. In the case of trypsins, after talisin intake, the larvae showed a 6-fold increase in Try6 gene expression, whereas two other genes were less expressed (Figure 7A). Regarding chymotrypsin genes, the Chy21 gene had its expression increased by 4-fold, and the Chy2 gene was less expressed than in control-fed larvae (Figure 7B). There is evidence to suggest that some insects are able to modulate the set of serinepeptidases according to the type of inhibitor ingested through differential regulation of the trypsin and chymotrypsin genes [31,36,39]. Similar phenomenon is also observed in coleopteran to overcome plant defensive cysteine protease inhibitor [35]. The expression profile of trypsins and chymotrypsins after ingestion of talisin differs from the expression profile observed in other studies with *S. frugiperda* fed on a diet containing soybean peptidase inhibitors [40]. Souza, Dias, Castelhano, Brandão, Moura and Silva-Filho [40] reported that the ingestion of soybean peptidase inhibitors by *S. frugiperda* resulted in the activation of a series of serinepeptidase genes, being able to distinguish one group of responsive genes and the other of genes that were not responsive to the inhibitor. As mentioned previously, we observed in this work that, after chronic ingestion of talisin, only one trypsin gene was overexpressed and two trypsin genes were less expressed; and there was one overexpressed chymotrypsin gene and one less expressed.

Based on the gene expression results, we aimed to establish a relationship between the binding affinities of talisin for the more and the less expressed enzymes, at atomic level. Since the purification of all enzyme isoforms would not be possible, we carried out in silico molecular docking simulations, an appropriate technique for this type of study. Freire, Franco, Kubo, Migliolo, Vargas, de Oliveira, Parra and Macedo [9] showed that the mechanism of talisin inhibition is a non-competitive type, which was also observed in our work for all talisin/serine protease complexes. This mechanism of inhibition is known to block the subtract access to the trypsin/chymotrypsin active site. Also in parallel with previously reported data [9], no direct reactions with the trypsin/chymotrypsin catalytic site were observed in our study. Similar computational finding have also been reported for Kunitz-type trypsin inhibitors from *Adenantera pavonina* (ApKTI) [22,41]. However, differently from ApKTI, talisin presents an important substitution at position 64 (arginine in ApKTI and glutamic acid in talisin). This amino acid substitution compromise the function of the so-called “reactive site loop”, which is characteristic of trypsin inhibitors [42]. Therefore, it is expected that talisin inhibits serine proteases by means of inhibitor/protease interactions that does not strongly rely on this reactive loop, as observed in the present study. Our results show that talisin establishes a higher number of interactions with the less expressed trypsin (Try12) when compared with the more expressed trypsin (Try 6) (Table 4). The same pattern is observed for the differently expressed chymotrypsins here reported (Table 5). Moreover, the type of interactions, including hydrogen bonds and hydrophobic interactions, as well as the average distance between all atoms involved in interactions are in agreement with those reported by Freire, Franco, Kubo, Migliolo, Vargas, de Oliveira, Parra and Macedo [9]. Thus, in general, we may conclude that because of the greater number of interactions and higher binding affinity with Try12 and Chy2, these enzymes are more sensitive to talisin, supporting our in vitro data. It could also be inferred for the complexes talisin/Try6 and talisin/Chy21, which presented fewer interactions and lower binding affinities, suggesting that talisin does not significantly interfere with the physiological function of these enzymes, which were overexpressed in the real-time PCR analyses.

Insects have several groups of peptidases comprising their digestive system. These peptidases have high rates of self-activation, which allows the insect to have high transcription diversity. This feature represents an acquired evolutionary advantage due to the need for rapid digestion, exploration of several types of food and, as a result of this, the need to avoid negative and antinutritional effects of toxic plant proteins. The *Noctuideae* family has a high diversity of trypsin and chymotrypsin type enzymes, which may have resulted from these adaptation mechanisms [12]. While it is evident that insects are capable of expressing a wide variety of peptidases in response to exposure to peptidase inhibitors, the mechanism of this induction is still not clearly known. The evolutionary relationship between biologically inactive proteins or sparing active proteins and normally active enzymes or bioactive proteins strongly suggests that some reserve proteins may be derived from genes that originally encoded proteins with a well-defined enzymatic action or other biological activity [43]. In consequence, some carbohydrate binding proteins function as multifunctional molecules, and may show sequence homology with Kunitz inhibitors, without, however, having trypsin inhibitory activity or being less active. On the contrary, they exhibit lectin-like activities [44,45,46].

Since talisin is a protein that binds strongly to chitin [47], and aiming to explore the specific carbohydrate interaction properties that talisin presents, we performed morphological analyses of the midgut of the talisin-fed larvae and analyzed two other classes of enzymes involved in the digestive process of *S. frugiperda*, α-amylase and aminopeptidase, enzymes involved in the digestion of carbohydrates and final digestion of peptides, respectively [38]. We observed no change in α-amylase activity, but we found that the aminopeptidase activity of intestinal lumen extracts was substantially higher in talisin-fed larvae when compared to the control and even to the epithelium. The activity of the enzymes isolated from the larvae’s intestinal epithelium from the group fed with talisin was also higher. Aminopeptidases are microvillary enzymes typically anchored to the intestinal epithelium [48]. The increase of aminopeptidase activity in the lumen of talisin-fed larvae possibly occurs due to epithelial damage and/or peritrophic membrane rupture (Figure 6). In addition, we did not observe increased expression of any aminopeptidase gene. 

In Lepidoptera, the midgut is surrounded by a semipermeable structure composed of microfibrils of chitin associated with proteoglycans and glycoproteins, called the peritrophic membrane [38,49]. The peritrophic membrane acts as a physical and chemical barrier that compartmentalizes the digestive process in insects, favoring high digestive efficiency as well as the recycling of digestive enzymes [50]. After initial digestion of food into the endoperitrophic space, molecules become sufficiently small and cross the pores of the peritrophic membrane into the ectoperitrophic space (accompanied by polymer hydrolases), and then flow into the cecum and the anterior intestine, where the intermediate and final digestion occurs by the enzymes anchored on the surface of the intestinal epithelium.

A large enzyme (e.g. aminopeptidase molecular mass between 90 and 130 kDa) has a larger diameter than the peritrophic membrane pores of *S. frugiperda* [51]. Therefore, in normal physiological situations it must be found in the ectoperitrophic space [38]. The presence of aminopeptidase in the endoperitrophic space suggests that the peritrophic membrane had its integrity compromised. Morphological analyses showed changes in both the epithelium and impairment of peritrophic membrane integrity in talisin-fed larvae (Figure 6). Molecules with a tendency to bind to chitin may compete for the binding sites of the endogenous chitin binding proteins, dissociating the normal structure of the membrane-protein complexes and finally modifying the physiology of the larval digestive tract. The disruption of the peritrophic membrane adversely affects the insect’s development by decreasing digestive efficiency, as well as increasing the metabolic costs associated with the synthesis of new enzymes by disruption of the enzymatic recycling mechanism [23,52,53,54,55].

Exploring proteins that are multifunctional, including talisin, may be an advantageous path in the search for molecules that can be used in more efficient strategies for pest control. The pyramiding of multiple defense genes in a plant is a promising strategy to increase its protection in order to avoid or delay the development of insect pest resistance [14,56]. The use of entomotoxic genes [57,58] is only one of the existing options for pest control. The more we understand the effects of plant defense proteins, as well as the adaptive responses used by insects, the more we will move in the right direction for efficient and ecologically appropriate choices, and also make it possible to integrate these with other pest control techniques.

## 4. Material and Methods

### 4.1. Talisin Extraction and Purification

*T. esculenta* seeds were obtained from the seed bank of the Laboratory of Protein Purification and Biological Functions of the Universidade Federal de Mato Grosso do Sul, Campo Grande, Brazil, and purified as previously reported by Freire et al. [9]. *T. esculenta* seeds were finely ground, defatted with hexane and extracted (meal to buffer ratio of 1:5) with 150 mM NaCl for 24 h at 4 °C and then centrifuged at 10,000 *g* for 30 min at the same temperature. The clear supernatant (crude extract or CE) was used to determine the protein content. The CE was diluted in 150 mM NaCl and applied to a Sephadex G-100 column (2.5 cm × 80 cm) equilibrated with the same solution. The protein-rich fraction was recovered and applied to a chitin column (1.5 cm × 10 cm) equilibrated with 50 mM phosphate buffer, pH 7.6, and eluted with 100 mM HCl. The purified protein was dialyzed and lyophilized.

### 4.2. Insects 

The colony of *S. frugiperda* (J.E. Smith, 1797) (Lepidoptera, Noctuidae) was maintained in standard conditions (27 ± 1 °C, 60–70% relative humidity and a 14:10 light-to-dark photoperiod) and fed on an artificial diet composed of jack bean, wheat germ, soybean flour, casein, vitamin complex, ascorbic acid, agar, formaldehyde and microbial inhibitors (tetracycline, sorbic acid and nipagin) [59].

### 4.3. In Vivo Insect Assays

To evaluate the effects of talisin on *S. frugiperda* development, neonate larvae were selected and individually transferred to glass tubes containing artificial diet supplemented with talisin at a concentration of 0.1%, 0.5% or 1% (*w*/*w*) until reaching the fifth instar. Control larvae were fed a diet devoid of talisin. Each treatment was composed of twenty larvae, and the experimental results are the average of three independent bioassays. Larval mass and survival were determined when the larvae reached the fifth instar under standard conditions. The treatment that was found to reduce larval mass by 50% relative to controls was selected for the subsequent enzymatic assays and molecular analysis. In the same treatment group, pupal mass, pupal stage and the number of emerging adults were counted to determine the mortality (M), and the time elapsed until adult emergence was recorded to estimate mean development time (T).

### 4.4. Midgut and Frass Preparation

Fifth-instar larvae were cold-immobilized and their midguts dissected in cold 0.15 M NaCl. Two types of samples of the midguts were prepared (I) whole midgut homogenates (midgut epithelium + midgut contents) and (II) midgut epithelium homogenates. The frass of *S. frugiperda* were separated from the rest of the tube’s diet and then collected. Both the samples of midguts and the frass were homogenized in cold 0.15 M NaCl with a handheld Potter-Elvehjem homogenizer. After homogenizing, the samples were centrifuged at 14,000× *g* at 4 °C for 20 min, and the supernatants were stored at −20 °C until their use as an enzyme source.

### 4.5. Nutritional Parameters

The dry weight of the larvae at maximum development, food consumed and feces eliminated were measured to determine the nutritional parameters (*n* = 20). Consumption, digestion, and food utilization indices were calculated according to the methodology proposed by Waldbauer [60] and modified by Scriber and Slansky Jr [61]. Weight of ingested food during *T* (*I*), larval weight gain during *T* (*B*), mean larval weight during *T* ($\bar{B}$), weight of frass produced during *T* (*F*), and duration of feeding period (*T*) were employed to determine the following parameters: efficiency of conversion of ingested food (ECI), calculated as (*B/I*) × 100, expressing the percentage of ingested food actually converted to biomass; efficiency of conversion of digested food (ECD), calculated as [*B*/(*I − F*)] × 100, expressing the efficiency with which digested food is converted to biomass; Relative consumption ratio (RCR) calculated as *I*/($\bar{B}$ *× T*), expressing the amount of ingested food per milligram of insect’s body weight per day; Relative growth ratio (RGR) calculated as *B*/*(*$\bar{B}$ *× T*), indicating the biomass gain by the insect in relation to its weight; Relative metabolic ratio (RMR), calculated as *M*/($\bar{B}$ *× T*), indicating the amount of food spent on metabolism per milligram of body weight and approximate digestibility (AD), calculated as [(*I − F*)*/I*] × 100, expressing the amount of ingested food that undergoes digestion. Metabolic cost (MC) was calculated as 100 − [*B*/(*I − F*)] × 100.

### 4.6. Protein Quantification

Protein concentration was determined according to Bradford [62], using bovine serum albumin (BSA) as a standard.

### 4.7. Enzymatic Assays

Trypsin-like and chymotrypsin-like activities were determined according to Oliveira, et al. [63]. The activity of trypsin-like enzymes was assayed using *N*-benzoyl-DL-arginyl-*p*-nitroanilide (BApNA; Sigma-Aldrich, St. Louis, MO, USA) as a model substrate. A sample (10 µL) of midgut juice extract was mixed with 60 µL of 50 mM Tris-HCl, pH 8.0 for 15 min, followed by 200 µL of BApNA for 30 min. Chymotrypsin-like activity was assayed using *N*-succinyl-alanine-alanine-proline-phenylalanine *p*-nitroanilide (SAAPFpNA; Sigma-Aldrich, St. Louis, MO, USA) as a model substrate. A sample of midgut juice extract (10 µL) was mixed with 90 µL of 50 mM Tris-HCl, pH 8.0 for 15 min, followed by 20 µL of SAAPFpNA for 5 min. All substrates were used at a final concentration of 1 mM. All assays were carried out at 30 °C. Absorbance was read at 405 nm. One unit of an enzyme (U) is defined as the amount that hydrolyzes 1 µmol of substrate per minute.

To analyze the sensitivity of trypsin-like enzymes in the midgut to inhibition by talisin, an inhibition curve was made with increasing concentrations of talisin (0–0.7 µg), and the trypsin activity was measured as described above.

α-Amylase activity was determined by employing a 3,5-dinitrosalicylic acid (DNS; Sigma-Aldrich) reagent prepared according to Noelting and Bernfeld [64]. An increase in reducing power, measured by the DNS reagent, was used as a measure of starch digestion. Aliquots (25 µL) of midgut juice extract were incubated with 25 µL of substrate–buffer solution (1% potato soluble starch in 50 mM CAPS buffer at pH 9.6 containing 2 mM CaCl_2_ and 20 mM NaCl). The reaction was stopped by the addition of 200 µL DNS. The resulting solution was heated in a boiling water bath for 5 min and cooled. After the addition of 200 µL of distilled water, absorbance was read at 550 nm. One enzyme unit (U) is defined as the amount of enzyme that produces 1 µmol of maltose equivalent per minute. 

The enzymatic assays for aminopeptidase N detection were performed using the synthetic substrate leucine p-nitroanilide (LpNa; Sigma-Aldrich) at a concentration of 1 mM, as described by Erlanger, et al. [65]. The reaction volume comprised 10 µL of midgut epithelium homogenates or midgut juice extract and 40 µL of the substrate in 50 mM Tris buffer, pH 7.5 at 30 °C for 30 min. The reaction was interrupted by adding 50 μL of 30% acetic acid. Absorbance was read at 410 nm. Three independent experiments were run in triplicate for each assay.

### 4.8. Peptidase Activity of Midgut Juice Extract in Native Polyacrylamide Gel Containing 1% Casein

Native slab polyacrylamide gel electrophoresis (PAGE) was used to separate peptidases on discontinuous polyacrylamide gel (4% stacking gel and 8% resolving gel), as described by Hivrale, Lomate, Basaiyye and Kalve [37], with a few modifications. Enzyme activity was detected by zymography. Midgut juice extract (6 µg of protein) of larvae fed on 0.5% talisin and on the control diet were loaded on native PAGE. To allow the identification of trypsin-like enzymes, the samples were incubated with the synthetic trypsin inhibitor TLCK (1 mM *N*-*p*-tosyllysine chloroketone) at 30 °C for 30 min before being applied onto the gel. To evaluate whether talisin interferes in enzymatic activity on zymography, the samples were also incubated with talisin at 30 °C for 30 min before being applied onto the gel. Electrophoresis was performed at 20 mA. After electrophoresis, the gel was washed with distilled water and equilibrated in 0.1 M glycine–NaOH buffer at pH 9.6. After equilibration, the gel was placed in 1% (*w*/*v*) casein (prepared in the same buffer), incubated at 30 °C for 2 h, stained with Coomassie brilliant blue R-250, and destained for visualization of proteinase activity.

### 4.9. Talisin Digestion

To investigate talisin resistance to proteolysis, it was incubated with midgut juice extract of fifth-instar larvae (fed control and talisin-amended diets) in 50 mM Tris-HCl buffer, pH 8.0 for different periods. The talisin:midgut protein ratio was 1:5. Digestion was performed at 30 °C for 0.5, 2, 8, 16, and 24 h and interrupted by boiling the samples in water for 2 min. Degradation of BSA was used as a positive control for peptidase activity. The proteins were subsequently separated by SDS-PAGE (15%), as described by Laemmli [66], and then stained with 0.1% Coomassie brilliant blue R-250 for detection. The relative molecular masses of digestion products were estimated by SDS-PAGE using protein markers of known molecular mass.

### 4.10. Microscopy Analysis

For insect histopathology, sections of whole larvae from both control and talisin-fed larvae were examined. For light microscopy, whole larvae were fixed overnight in Bouin’s solution after several incisions had been made in the cuticle to allow the fixative to permeate the cuticle of the larvae. Larvae were then embedded in paraffin using a TP1020 Automatic Tissue Processor (Leica, Buffalo Grove, IL, USA). Sections were cut at 3–5 micrometer and then stained with haematoxylin/eosin and mounted on glass slides with DePeX mounting medium.

### 4.11. Quantitative Real-Time PCR

Total RNA was extracted from frozen midguts using 1 mL of TRIzol™ (Invitrogen, Carlsbad, CA, USA). The samples were treated with DNase I (Thermo Scientific, Pittsburgh, KS, USA) at 37 °C for 1 h. Synthesis of the cDNAs was primed by oligo d(T) using a High Capacity cDNA Reverse Transcription Kit (Applied Biosystems, Foster City, CA, USA) according to the manufacturer’s recommendations. The cDNA was diluted in a working solution and 8 μL was used in qRT-PCR (20 ng). The reactions were carried out in a thermocycler StepOne™ Real-Time PCR System (Applied Biosystems) using Maxima® SYBR Green/ROX qPCR Master Mix (2X) (Thermo Scientific, Pittsburgh, KS, USA). The master mix prepared for analysis of each gene was composed of 1 μL of forward primer (10 μM), 1 μL of reverse primer (10 μM), 10 μL of SYBR®, and 8 μL of cDNA (20 ng) in a total volume of 20 μL. The amplification reaction conditions were 95 °C for 10 min, 95 °C for 15 min followed by 40 cycles of 95 °C for 15 min, and 60 °C for 1 min. A negative control without a cDNA template was run with each analysis to evaluate the overall specificity. The ribosomal protein S30 and glyceraldehyde-3-phosphate dehydrogenase (GAPDH) were used to normalize the data (NCBI locus AF400225) [67]. The efficiency and specificity reaction of each primer set were evaluated by a standard curve and a melting curve. Relative quantification was carried out using REST® model with efficiency correction [68]. The experiments were repeated twice for validation of results.

### 4.12. Molecular Modeling

Molecular modeling studies were carried out with the primary sequences of talisin from *T. esculenta* (GenBank: ACJ51124.1), as well as trypsin (NCBI reference sequence: XP_022821647.1 and XP_022821658.1) and chymotrypsin (GenBank: ALO61082.1 and AIR09774.1) from *S. frugiperda*. Signal peptide and transmembrane topologies were predicted by the Phobius [69] server and disregarded for further analysis. Inactive precursor zymogens were also removed in order to obtain active sequences for the serine peptidases. Template structures for comparative modeling were identified through BLASTp [70] analysis. One hundred three-dimensional theoretical models were generated using MODELLER v 9.17 [71] using as template structures: (i) the crystal structure of the Kunitz (STI) type inhibitor from seeds of *Delonix regia* [72], for talisin; (ii) the crystal structure of a *Fusarium oxysporum* trypsin [73], for the trypsin; and (iii) the collagenase from the fly larvae *Hypoderma lineatum* [74], for chymotrypsin. All models were ranked according to their DOPE score (free energy). The lowest free energy models were validated regarding their stereochemistry and fold quality using the servers PROCHECK [15], ProSa-web [16] and Molprobity [17].

### 4.13. Molecular Docking

Once validated, the theoretical models for talisin, trypsin and chymotrypsin were used as inputs for molecular docking simulations in order to better understand the possible interactions occurring in the inhibitor/peptidases complexes. AutoDock Tools [75] was used to configure grid boxes of 60 × 60 × 60 points with 1 Å spacing and positioned at the center of the serine peptidases. The maximum freedom for the side chains from talisin was locked. Fifty runs of molecular docking simulations were carried out using AUTODOCK 4.2 [75], and the complexes ranked according to their binding affinities in kcal·mol^−1^. The best complex for each condition was then submitted to 50,000 steps of energy minimization (steepest descent) in cubic boxes filled with single point charge (SPC) water molecules using the GROMOS96 43a1 force field from the GROMACS 5.0.4 computational package [76]. Structural visualization and atomic interaction prediction (respecting the maximum distance of 3.6 Å) was done in PyMOL (https://pymol.org).

### 4.14. Statistical Analysis

The results were expressed as means ± standard error, where appropriate. Data on initial mortality rate, duration of larval and pupal periods, larval weight, nutritional parameters and enzymatic activity were subjected to one-way analysis of variance (ANOVA). When differences were found between treatments, Tukey’s test was applied to determine the level of significance (*p* < 0.05).

## Figures and Tables

**Table 1:** Effect of dietary 0.5% *Talisia esculenta* reserve protein (talisin) on *Spodoptera frugiperda* larval development.

Parameter	Control	0.5% Talisin
Pupal mass (mg)	275.08 ± 13.25 ^a^	272.86 ± 15.67 ^a^
Larval stage (days)	18.80 ± 0.97 ^a^	19.70 ± 0.78 ^b^
Pupal stage (days)	9.60 ± 0.91 ^a^	10.40 ± 1.01 ^a^
Adult life span (days)	9.66 ± 1.75 ^a^	8.33 ± 1.10 ^a^
Total development time (days)	35.70 ± 2.53 ^a^	39.00 ± 0.89 ^b^
Survival to adulthood (%)	100 ^a^	100 ^a^

Values are the means ± SE. Means on the same line with the same letters do not differ significantly (*p* < 0.05).

**Table 2:** Nutritional parameters in control and experimental (0.5% talisin-fed) fifth-instar *Spodoptera frugiperda* larvae.

Parameter	Control	0.5% Talisin
Relative Consumption Rate (g/g/day)	0.7490 ± 0.0801 ^a^	0.7646 ± 0.2592 ^a^
Relative Growth Ratio (g/g/day)	0.0833 ± 0.0060 ^a^	0.0809 ± 0.0102 ^a^
Relative Metabolic Ratio (g/g/day)	0.3907 ± 0.0613 ^a^	0.4605 ± 0.2018 ^a^
Approximate Digestibility (%)	63.526 ± 4.0253 ^a^	76.8555 ± 7.6049 ^b^
Efficiency of Conversion of Ingested Food (%)	11.966 ± 1.8135 ^a^	11.2151 ± 3.4421 ^a^
Efficiency of Conversion of Digested Food (%)	17.861 ± 2.6050 ^a^	14.8380 ± 5.8188 ^a^
Metabolic Cost (%)	85.447 ± 6.5185 ^a^	92.5912 ± 6.5681 ^a^

Values are the means ± SE. Means on the same line with the same letters do not differ significantly (*p* < 0.05).

**Table 3:** Structural statistics for the tridimensional theoretical models generated in this study for talisin, trypsin (6 and 12) and chymotrypsin (2 and 21).

Predicted Structures	Sequence Length	Fold Quality (Z-Score)	Stereochemistry (G-Factors)	Ramachandran Most Favored (%)	Ramachandran Allowed (%)	Ramachandran Outliers (%)	Bad Bonds (%)	Bad Angles (%)
Talisin	198	−4.62	−0.28	87.7	94.9	5.10	0.00	1.58
Trypsin 6	233	−5.82	−0.21	94.8	98.3	1.73	0.06	2.14
Trypsin 12	232	−5.28	−0.28	93.0	97.0	3.04	0.00	2.22
Chymotrypsin 2	234	−5.42	−0.25	94.8	97.8	2.16	0.00	1.68
Chymotrypsin 21	237	−5.82	−0.22	91.5	98.7	1.28	0.17	1.90

The z-scores obtained for all structures here reported are in agreement with those with similar size, structurally determined by X-ray crystallography and deposited in the Protein Data Bank (PDB). The G-factors indicate that the overall average for the dihedral angles, along with the main-chain covalent forces for each structure are within the expected values for reliable structures (G-factors > −0.5). The structural validations were performed on PROCHECK [15], ProSa-web [16] and MolProbity [17].

**Table 4:** In silico interactions for the complexes talisin/Trypsin 6 (−8.4 kcal·mol^−1^) and talisin/Trypsin 12 (−8.8 kcal·mol^−1^).

Residues	Positions	Atom Names	Distances (Å)	Residues	Positions	Atom Names	Interactions
Trypsin 6 (XP_022821647.1 *)				Talisin (ACJ51124.1 *)			
Asn	37	ND2	1.9	Ser	106	OG	HB
Ser	112	OG	3.1	Ser	50	N	HB
Ile	113	O	1.9	Gln	48	NE2	HB
Ile	113	N	2.5	Ser	49	OG	HB
Gly	115	N	3.1	Gln	48	NE2	HB
Ala	116	CB	3.6	Leu	34	CD2	H
Asn	197	O	2.1	Gln	53	NE2	HB
Ile	199	N	3.5	Gln	53	OE1	HB
Ile	199	N	3.3	Gln	53	NE2	HB
Ser	231	OG	1.9	Pro	35	O	HB
Trypsin 12 (XP_022821658 *)				Talisin (ACJ51124.1 *)			
Gly	4	O	2.9	Ile	117	O	HB
Thr	133	CG	3.6	Val	116	CG1	H
Thr	133	OG1	3.6	Ile	117	N	HB
Tyr	134	O	3.2	Tyr	65	OH	HB
Tyr	134	CE2	3.6	Met	126	CE	H
Tyr	135	OH	2.0	Tyr	65	O	HB
Ala	137	O	1.9	Tyr	65	OH	HB
Pro	138	O	3.3	Val	96	N	HB
Thr	139	O	1.7	Ser	94	OG	HB
Thr	139	OG1	2.3	Gln	82	OE1	HB
Thr	139	OG1	3.2	Val	96	N	HB
Ser	141	OG	3.4	Tyr	93	N	HB
Arg	145	NH2	2.3	Ile	117	O	HB
Arg	145	NE	2.7	Ile	117	O	HB

Å: Ångström; HB: Hydrogen bond; H: hydrophobic interactions; * NCBI identification.

**Table 5:** In silico interactions for the complexes talisin/Chymotrypsin 2 (−11.2 kcal·mol^−1^) and talisin/Chymotrypsin 12 (−10.0 kcal·mol^−1^).

Chymotrypsin 2 (ALO61082.1 *)			Distances (Å)	Talisin (ACJ51124.1 *)			Interactions
Residues	Positions	Atom Names		Residues	Positions	Atom Names	
Ile	35	O	2.6	Asn	66	OD1	HB
Ile	35	O	3.2	Asn	66	ND2	HB
His	81	CE1	3.5	Val	116	CG1	H
Ile	110	O	2.8	Asn	66	ND2	HB
Leu	112	N	3.2	Tyr	65	O	HB
Asn	119	OD1	3.1	Val	149	N	H
Asn	119	OD1	3.2	Ser	148	OG	HB
Leu	201	CD2	3.6	Tyr	65	CE1	H
Ser	229	OG	2.9	Lys	98	NZ	HB
Gln	232	OE1	2.9	Val	63	O	HB
Gln	232	OE1	3.2	Asn	80	OD1	HB
Ser	233	O	3.4	Ser	94	OG	HB
Ser	233	OG	3.5	Gln	82	OE1	HB
Gln	234	O	2.9	Gln	82	ND2	HB
Gln	234	OE1	2.9	Tyr	93	OH	HB
Gln	234	NE2	3.0	Tyr	93	OH	HB
Trypsin 21 (AIR09774.1 *)				Talisin (ACJ51124.1 *)			
Leu	9	N	3.0	Asp	70	OD2	HB
Glu	11	OE1	3.6	Gly	67	N	HB
Gln	102	NE2	3.3	Asp	192	OD2	HB
Phe	103	O	3.0	Asp	192	N	HB
Ala	111	CB	3.3	Tyr	93	CD1	H
Leu	112	O	2.9	Tyr	93	OH	HB
Ser	116	OG	3.5	Gln	92	OE1	HB
Gln	117	OE1	2.9	Tyr	93	N	HB
Tyr	151	OH	3.6	Asn	66	O	HB
Gln	197	NE2	3.1	Asn	66	ND2	HB
Gln	197	OE1	3.6	Asn	66	ND2	HB
Arg	198	NH2	3.4	Tyr	65	O	HB
Arg	198	NH2	3.5	Asn	66	O	HB
Gly	200	O	3.6	Tyr	65	OH	HB

Å: Ångström; HB: Hydrogen bond; H: hydrophobic interactions; * NCBI identification.
